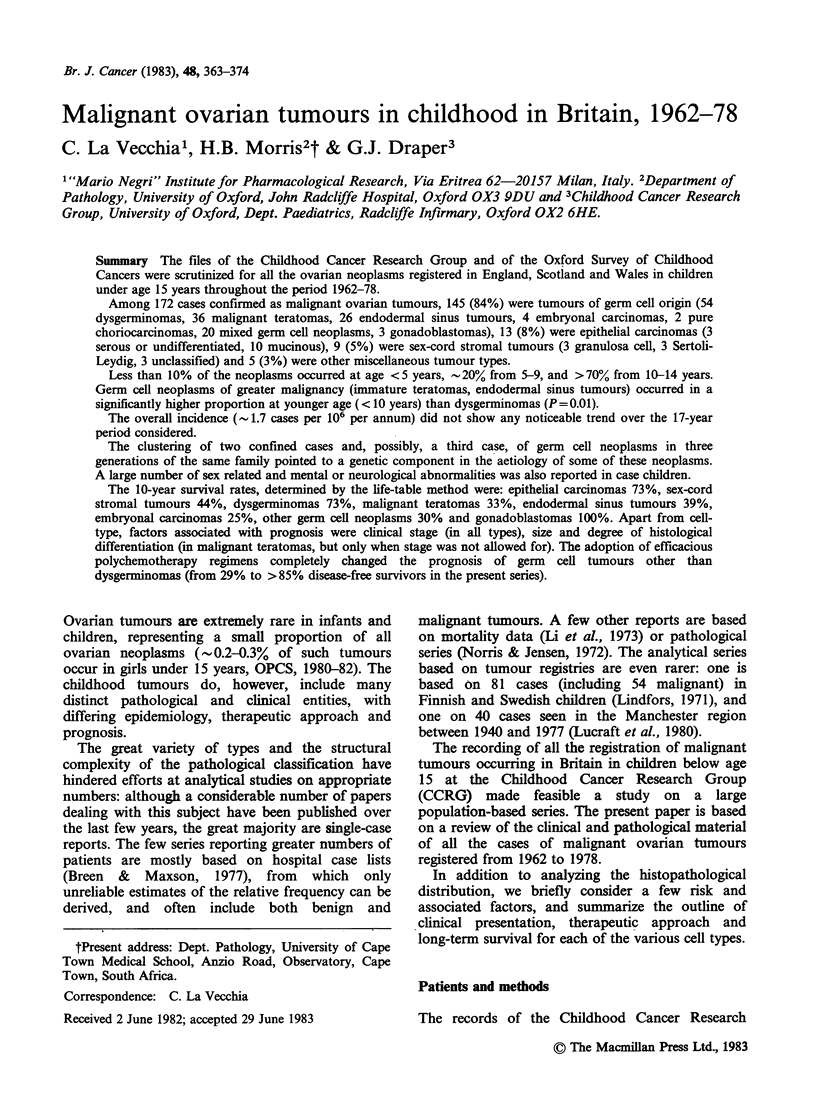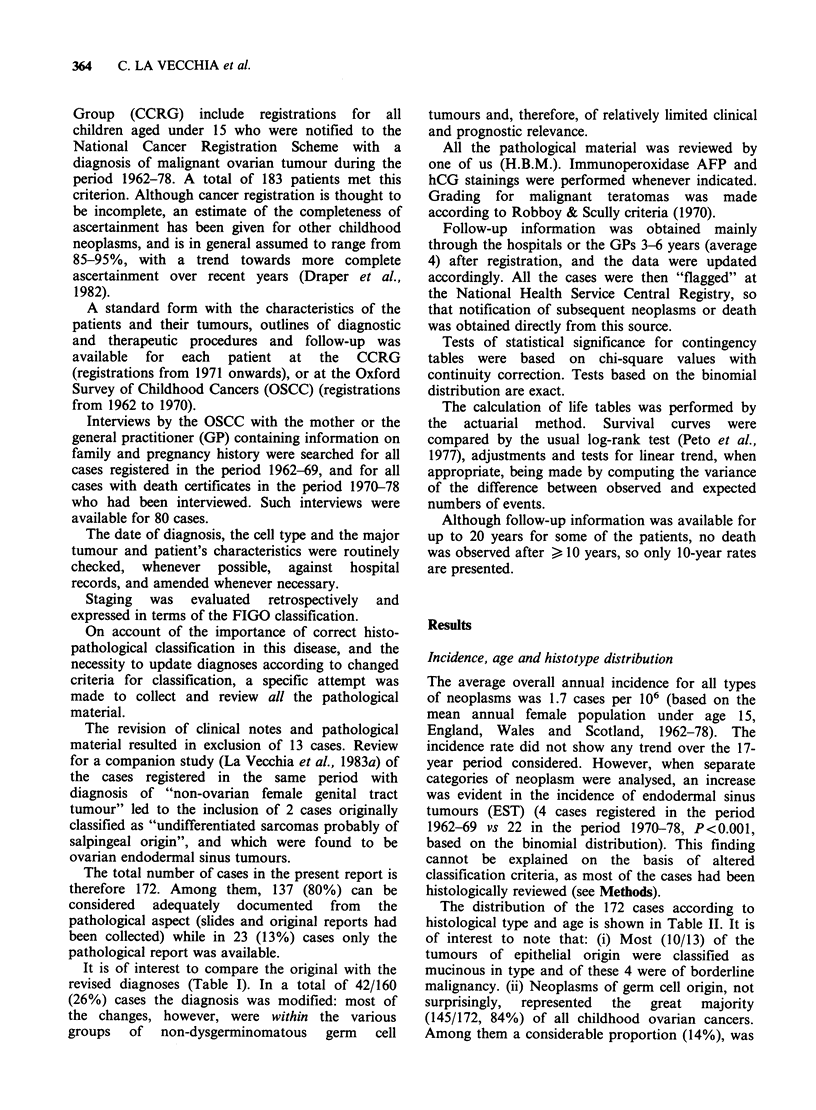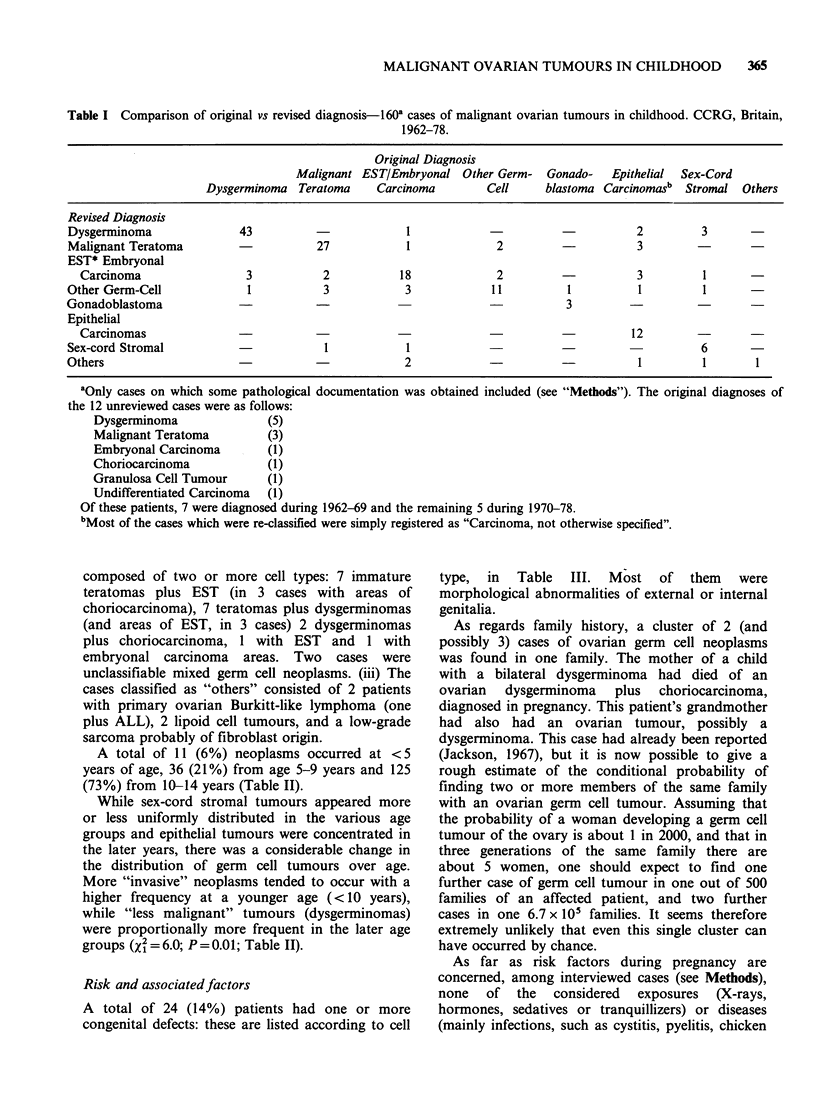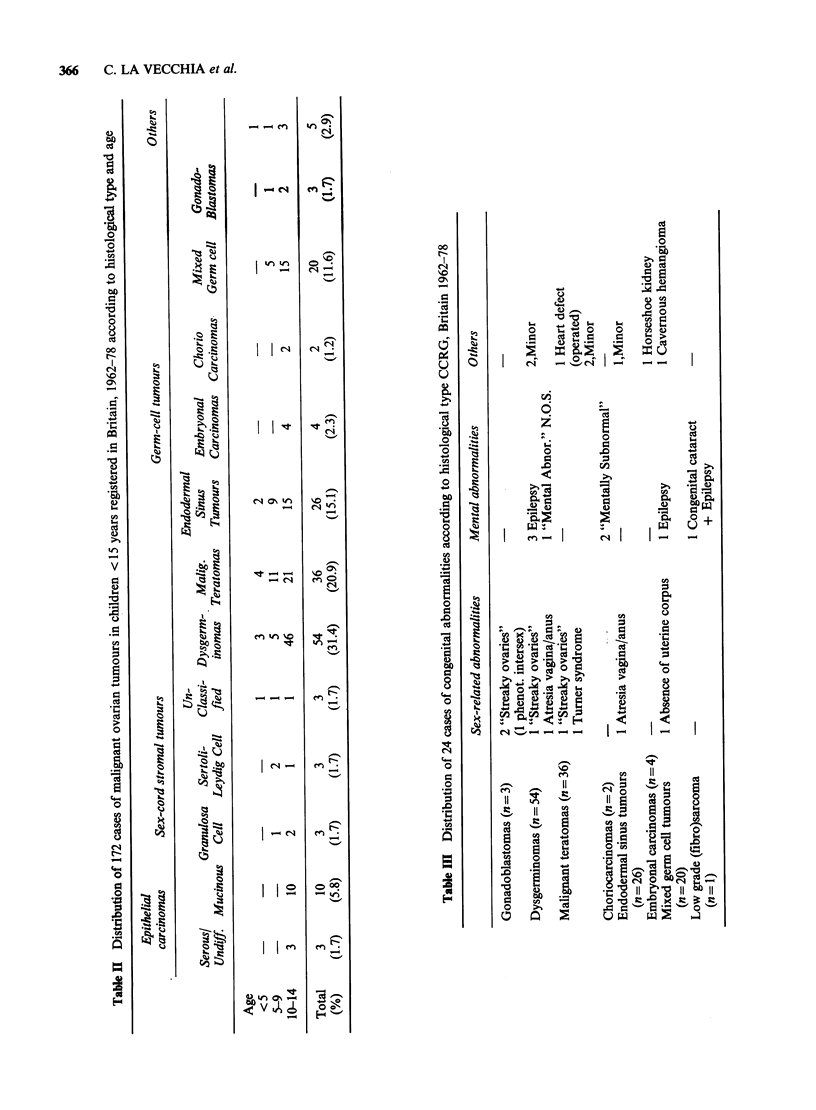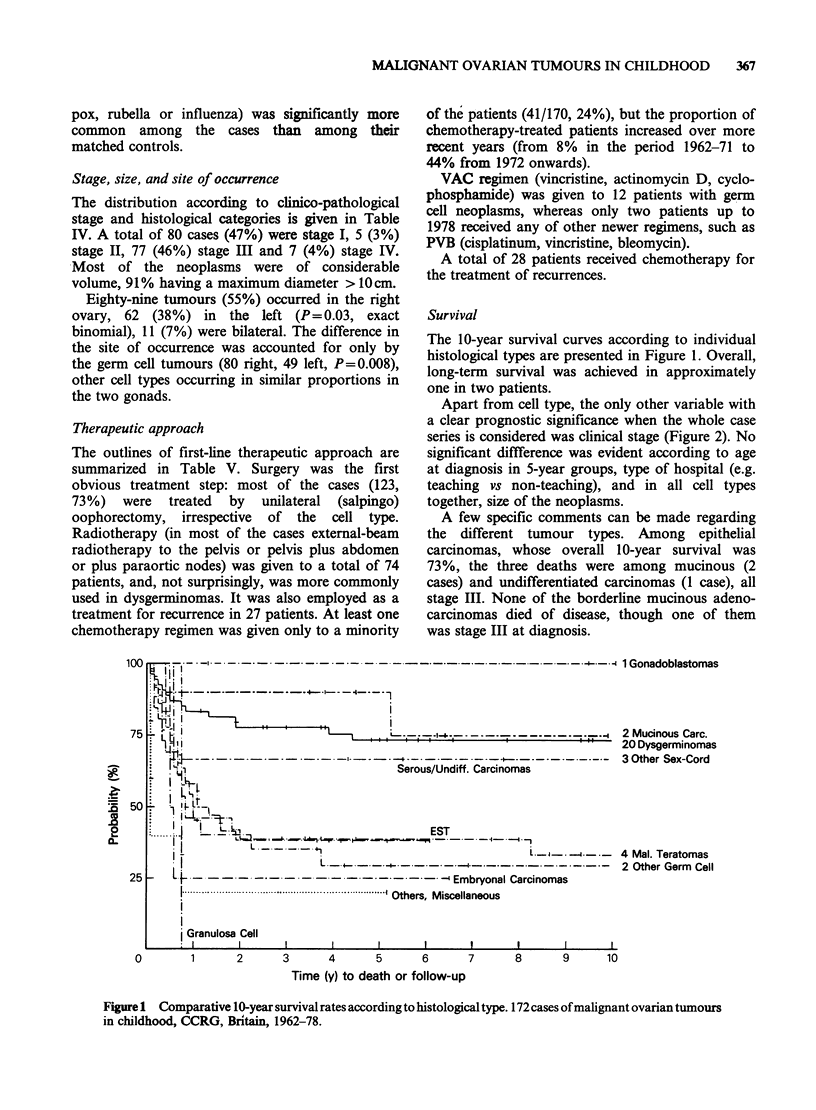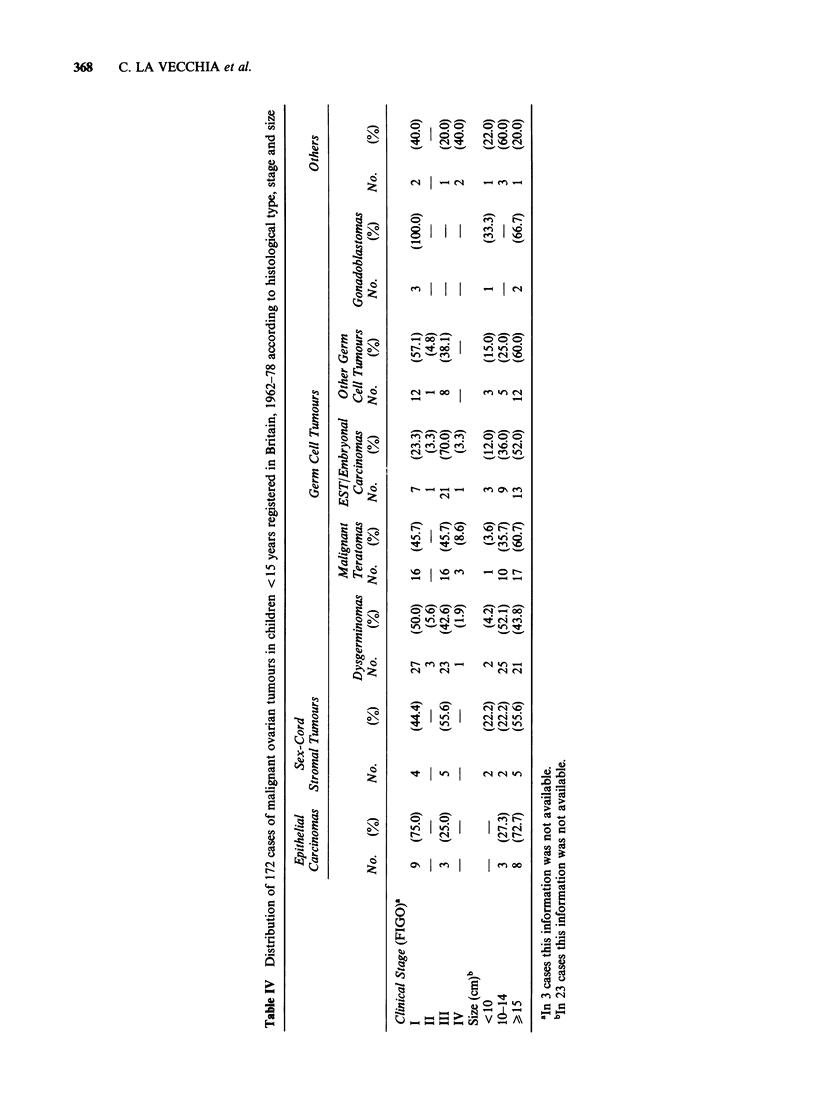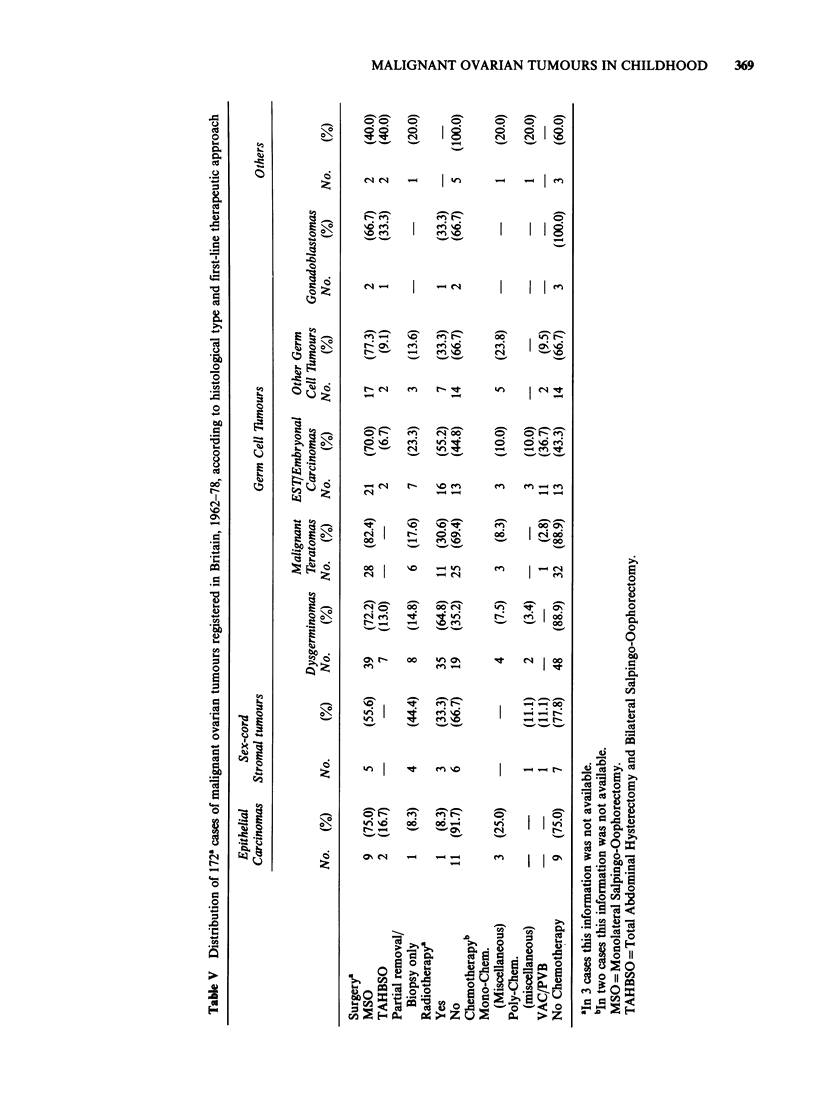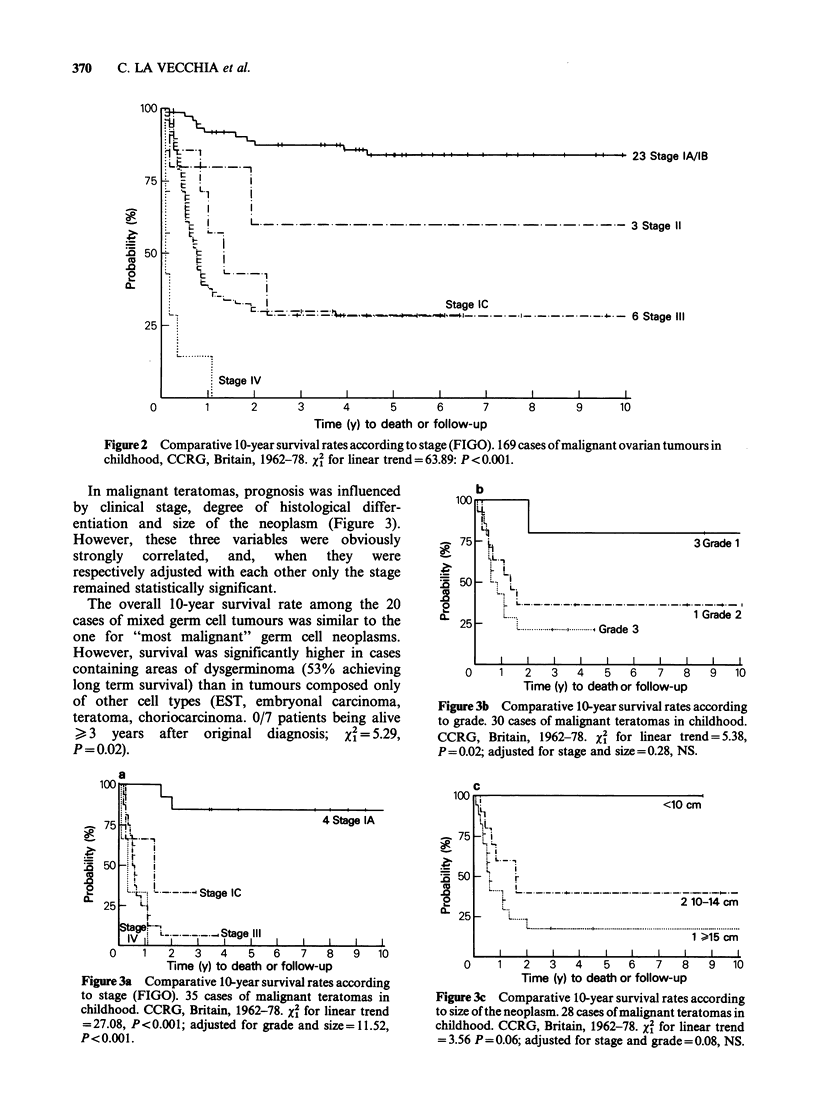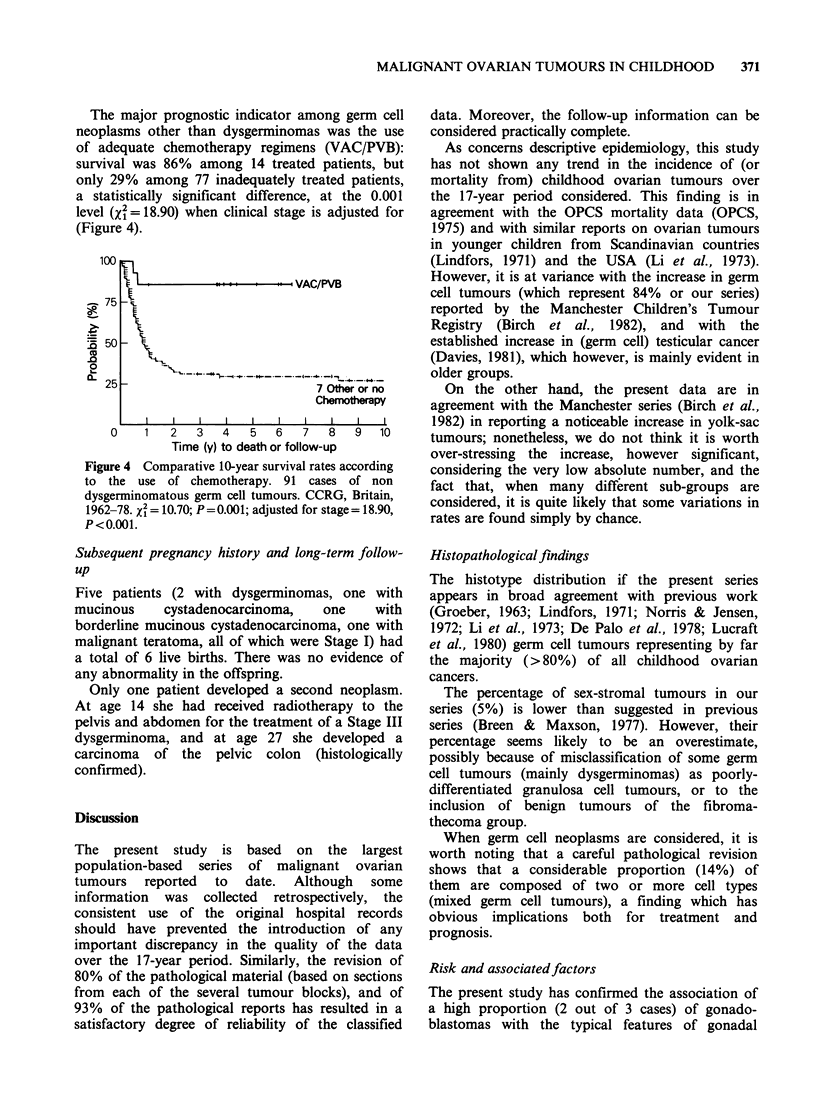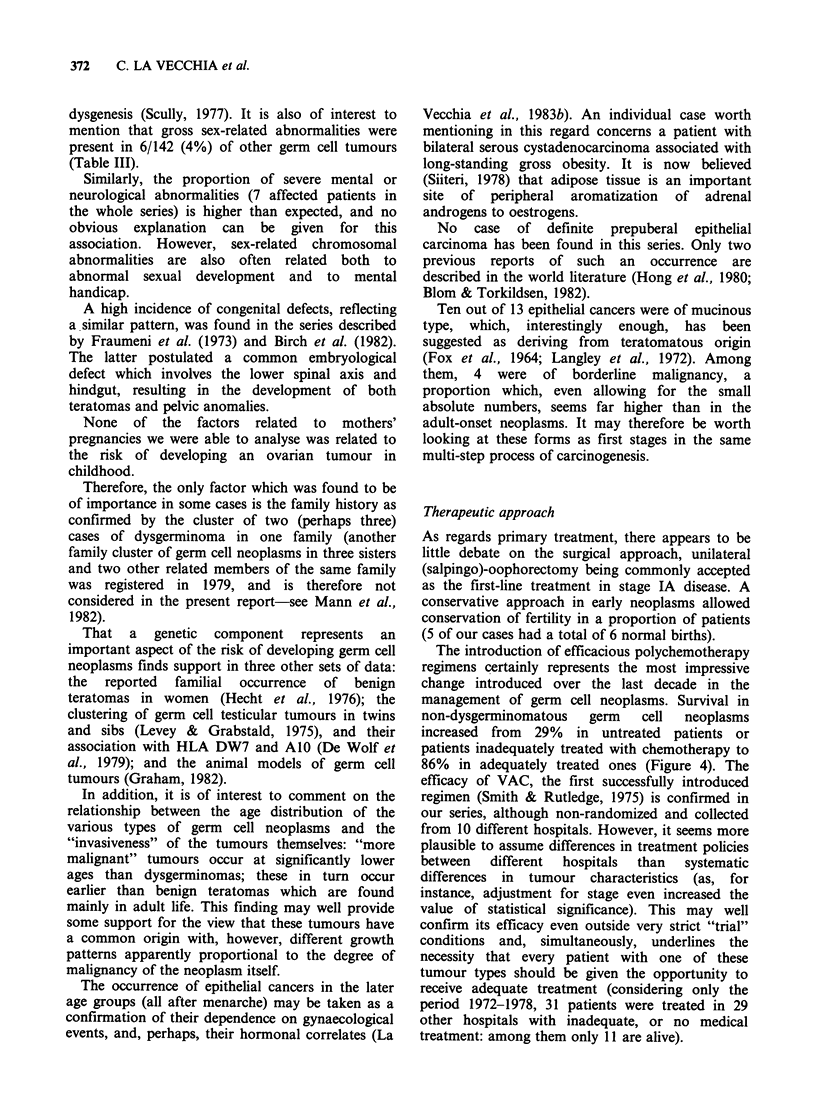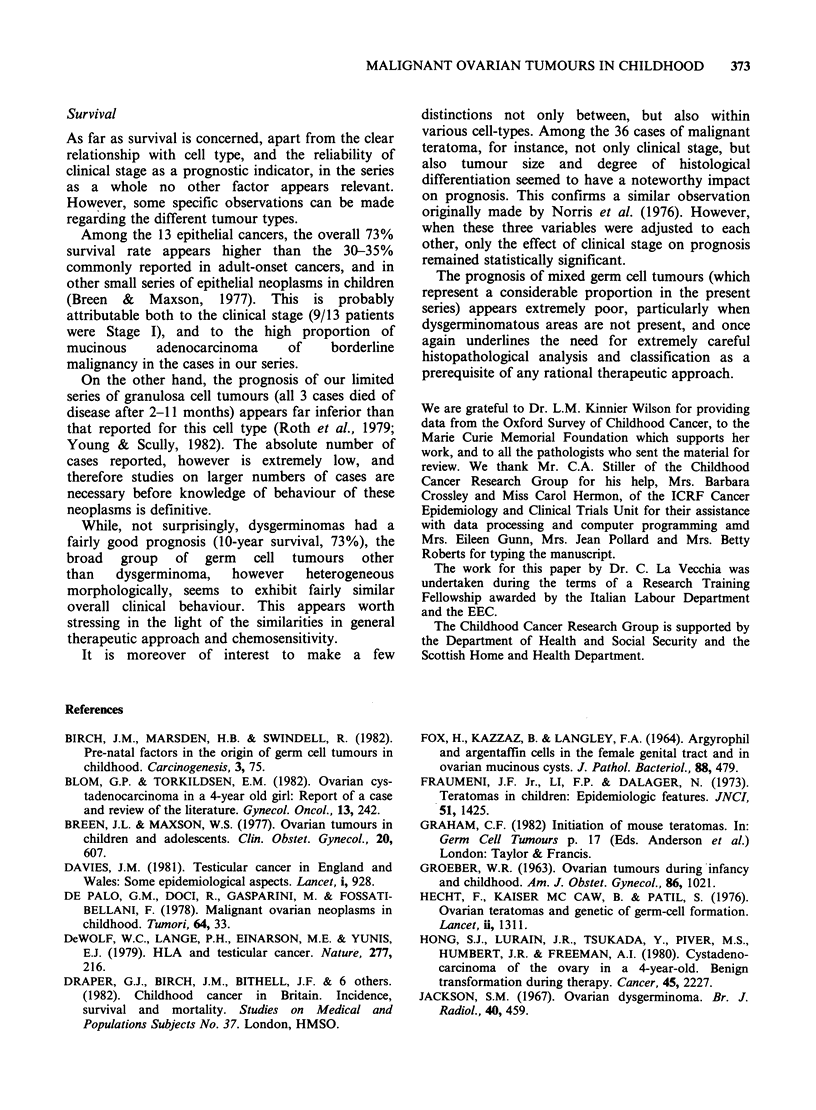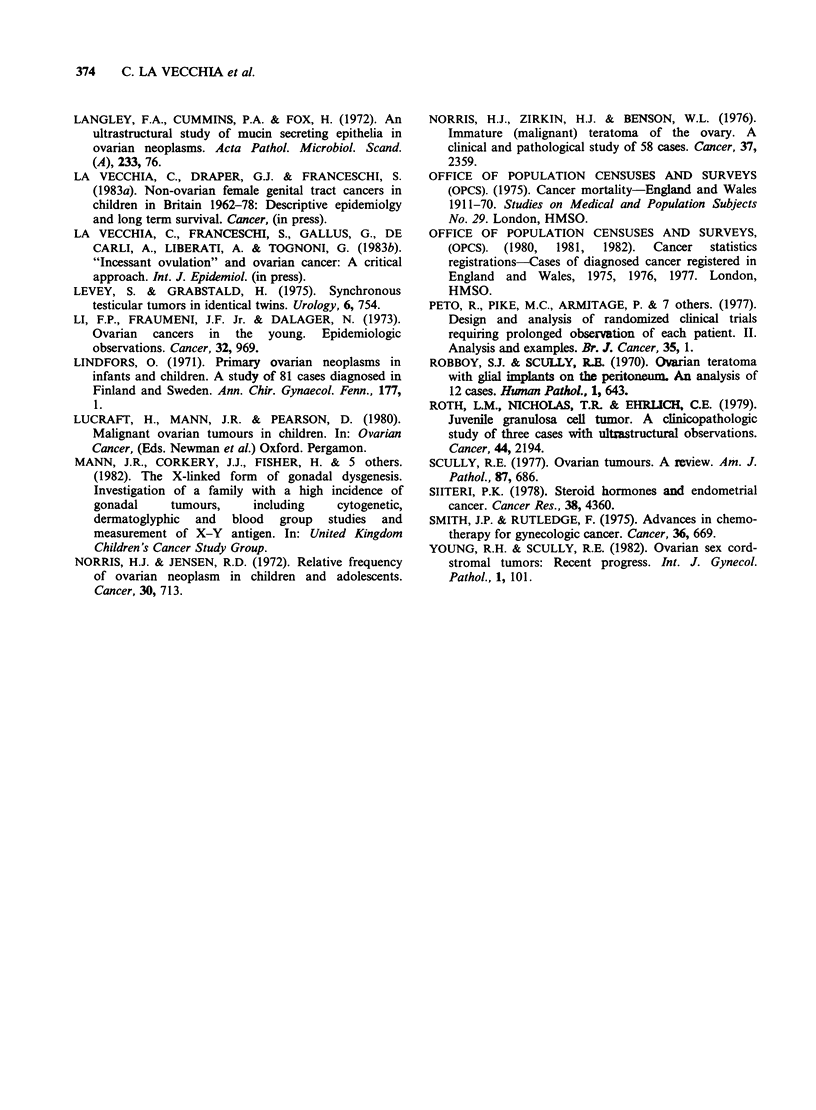# Malignant ovarian tumours in childhood in Britain, 1962-78.

**DOI:** 10.1038/bjc.1983.201

**Published:** 1983-09

**Authors:** C. La Vecchia, H. B. Morris, G. J. Draper

## Abstract

The files of the Childhood Cancer Research Group and of the Oxford Survey of Childhood Cancers were scrutinized for all the ovarian neoplasms registered in England, Scotland and Wales in children under age 15 years throughout the period 1962-78. Among 172 cases confirmed as malignant ovarian tumours, 145 (84%) were tumours of germ cell origin (54 dysgerminomas, 36 malignant teratomas, 26 endodermal sinus tumours, 4 embryonal carcinomas, 2 pure choriocarcinomas, 20 mixed germ cell neoplasms, 3 gonadoblastomas), 13 (8%) were epithelial carcinomas (3 serous or undifferentiated, 10 mucinous), 9 (5%) were sex-cord stromal tumours (3 granulosa cell, 3 Sertoli-Leydig, 3 unclassified) and 5 (3%) were other miscellaneous tumour types. Less than 10% of the neoplasms occurred at age less than 5 years, approximately 20% from 5-9, and greater than 70% from 10-14 years. Germ cell neoplasms of greater malignancy (immature teratomas, endodermal sinus tumours) occurred in a significantly higher proportion at younger age (less than 10 years) than dysgerminomas (P = 0.01). The overall incidence (approximately 1.7 cases per 10(6) per annum) did not show any noticeable trend over the 17-year period considered. The clustering of two confined cases and, possibly, a third case, of germ cell neoplasms in three generations of the same family pointed to a genetic component in the aetiology of some of these neoplasms. A large number of sex related and mental or neurological abnormalities was also reported in case children. The 10-year survival rates, determined by the life-table method were: epithelial carcinomas 73%, sex-cord stromal tumours 44%, dysgerminomas 73%, malignant teratomas 33%, endodermal sinus tumours 39%, embryonal carcinomas 25%, other germ cell neoplasms 30% and gonadoblastomas 100%. Apart from cell-type, factors associated with prognosis were clinical stage (in all types), size and degree of histological differentiation (in malignant teratomas, but only when stage was not allowed for). The adoption of efficacious polychemotherapy regimens completely changed the prognosis of germ cell tumours other than dysgerminomas (from 29% to greater than 85% disease-free survivors in the present series).


Br. J. Cancer (1983), 48, 363–374

### Malignant ovarian tumours in childhood in Britain, 1962–78

C. La Vecchia[1], H.B. Morris[2]† & G.J. Draper[3]

[1]*"Mario Negri" Institute for Pharmacological Research, Via Eritrea 62—20157 Milan, Italy.* [2]*Department of Pathology, University of Oxford, John Radcliffe Hospital, Oxford OX3 9DU and* [3]*Childhood Cancer Research Group, University of Oxford, Dept. Paediatrics, Radcliffe Infirmary, Oxford OX2 6HE.*

**Summary** The files of the Childhood Cancer Research Group and of the Oxford Survey of Childhood Cancers were scrutinized for all the ovarian neoplasms registered in England, Scotland and Wales in children under age 15 years throughout the period 1962–78.

Among 172 cases confirmed as malignant ovarian tumours, 145 (84%) were tumours of germ cell origin (54 dysgerminomas, 36 malignant teratomas, 26 endodermal sinus tumours, 4 embryonal carcinomas, 2 pure choriocarcinomas, 20 mixed germ cell neoplasms, 3 gonadoblastomas), 13 (8%) were epithelial carcinomas (3 serous or undifferentiated, 10 mucinous), 9 (5%) were sex-cord stromal tumours (3 granulosa cell, 3 Sertoli-Leydig, 3 unclassified) and 5 (3%) were other miscellaneous tumour types.

Less than 10% of the neoplasms occurred at age $<5$ years, $\sim 20\%$ from 5–9, and $>70\%$ from 10–14 years. Germ cell neoplasms of greater malignancy (immature teratomas, endodermal sinus tumours) occurred in a significantly higher proportion at younger age ($<10$ years) than dysgerminomas ($P=0.01$).

The overall incidence ($\sim 1.7$ cases per $10^6$ per annum) did not show any noticeable trend over the 17-year period considered.

The clustering of two confined cases and, possibly, a third case, of germ cell neoplasms in three generations of the same family pointed to a genetic component in the aetiology of some of these neoplasms. A large number of sex related and mental or neurological abnormalities was also reported in case children.

The 10-year survival rates, determined by the life-table method were: epithelial carcinomas 73%, sex-cord stromal tumours 44%, dysgerminomas 73%, malignant teratomas 33%, endodermal sinus tumours 39%, embryonal carcinomas 25%, other germ cell neoplasms 30% and gonadoblastomas 100%. Apart from cell-type, factors associated with prognosis were clinical stage (in all types), size and degree of histological differentiation (in malignant teratomas, but only when stage was not allowed for). The adoption of efficacious polychemotherapy regimens completely changed the prognosis of germ cell tumours other than dysgerminomas (from 29% to $>85\%$ disease-free survivors in the present series).

Ovarian tumours are extremely rare in infants and children, representing a small proportion of all ovarian neoplasms ($\sim 0.2$–0.3% of such tumours occur in girls under 15 years, OPCS, 1980–82). The childhood tumours do, however, include many distinct pathological and clinical entities, with differing epidemiology, therapeutic approach and prognosis.

The great variety of types and the structural complexity of the pathological classification have hindered efforts at analytical studies on appropriate numbers: although a considerable number of papers dealing with this subject have been published over the last few years, the great majority are single-case reports. The few series reporting greater numbers of patients are mostly based on hospital case lists (Breen & Maxson, 1977), from which only unreliable estimates of the relative frequency can be derived, and often include both benign and malignant tumours. A few other reports are based on mortality data (Li *et al.*, 1973) or pathological series (Norris & Jensen, 1972). The analytical series based on tumour registries are even rarer: one is based on 81 cases (including 54 malignant) in Finnish and Swedish children (Lindfors, 1971), and one on 40 cases seen in the Manchester region between 1940 and 1977 (Lucraft *et al.*, 1980).

The recording of all the registration of malignant tumours occurring in Britain in children below age 15 at the Childhood Cancer Research Group (CCRG) made feasible a study on a large population-based series. The present paper is based on a review of the clinical and pathological material of all the cases of malignant ovarian tumours registered from 1962 to 1978.

In addition to analyzing the histopathological distribution, we briefly consider a few risk and associated factors, and summarize the outline of clinical presentation, therapeutic approach and long-term survival for each of the various cell types.

### Patients and methods

The records of the Childhood Cancer Research

†Present address: Dept. Pathology, University of Cape Town Medical School, Anzio Road, Observatory, Cape Town, South Africa.

Correspondence: C. La Vecchia

Received 2 June 1982; accepted 29 June 1983

© The Macmillan Press Ltd., 1983

Group (CCRG) include registrations for all children aged under 15 who were notified to the National Cancer Registration Scheme with a diagnosis of malignant ovarian tumour during the period 1962–78. A total of 183 patients met this criterion. Although cancer registration is thought to be incomplete, an estimate of the completeness of ascertainment has been given for other childhood neoplasms, and is in general assumed to range from 85–95%, with a trend towards more complete ascertainment over recent years (Draper *et al.*, 1982).

A standard form with the characteristics of the patients and their tumours, outlines of diagnostic and therapeutic procedures and follow-up was available for each patient at the CCRG (registrations from 1971 onwards), or at the Oxford Survey of Childhood Cancers (OSCC) (registrations from 1962 to 1970).

Interviews by the OSCC with the mother or the general practitioner (GP) containing information on family and pregnancy history were searched for all cases registered in the period 1962–69, and for all cases with death certificates in the period 1970–78 who had been interviewed. Such interviews were available for 80 cases.

The date of diagnosis, the cell type and the major tumour and patient's characteristics were routinely checked, whenever possible, against hospital records, and amended whenever necessary.

Staging was evaluated retrospectively and expressed in terms of the FIGO classification.

On account of the importance of correct histopathological classification in this disease, and the necessity to update diagnoses according to changed criteria for classification, a specific attempt was made to collect and review *all* the pathological material.

The revision of clinical notes and pathological material resulted in exclusion of 13 cases. Review for a companion study (La Vecchia *et al.*, 1983*a*) of the cases registered in the same period with diagnosis of "non-ovarian female genital tract tumour" led to the inclusion of 2 cases originally classified as "undifferentiated sarcomas probably of salpingeal origin", and which were found to be ovarian endodermal sinus tumours.

The total number of cases in the present report is therefore 172. Among them, 137 (80%) can be considered adequately documented from the pathological aspect (slides and original reports had been collected) while in 23 (13%) cases only the pathological report was available.

It is of interest to compare the original with the revised diagnoses (Table I). In a total of 42/160 (26%) cases the diagnosis was modified: most of the changes, however, were *within* the various groups of non-dysgerminomatous germ cell tumours and, therefore, of relatively limited clinical and prognostic relevance.

All the pathological material was reviewed by one of us (H.B.M.). Immunoperoxidase AFP and hCG stainings were performed whenever indicated. Grading for malignant teratomas was made according to Robboy & Scully criteria (1970).

Follow-up information was obtained mainly through the hospitals or the GPs 3–6 years (average 4) after registration, and the data were updated accordingly. All the cases were then "flagged" at the National Health Service Central Registry, so that notification of subsequent neoplasms or death was obtained directly from this source.

Tests of statistical significance for contingency tables were based on chi-square values with continuity correction. Tests based on the binomial distribution are exact.

The calculation of life tables was performed by the actuarial method. Survival curves were compared by the usual log-rank test (Peto *et al.*, 1977), adjustments and tests for linear trend, when appropriate, being made by computing the variance of the difference between observed and expected numbers of events.

Although follow-up information was available for up to 20 years for some of the patients, no death was observed after $\geqslant 10$ years, so only 10-year rates are presented.

### Results

### *Incidence, age and histotype distribution*

The average overall annual incidence for all types of neoplasms was 1.7 cases per $10^6$ (based on the mean annual female population under age 15, England, Wales and Scotland, 1962–78). The incidence rate did not show any trend over the 17-year period considered. However, when separate categories of neoplasm were analysed, an increase was evident in the incidence of endodermal sinus tumours (EST) (4 cases registered in the period 1962–69 *vs* 22 in the period 1970–78, $P<0.001$, based on the binomial distribution). This finding cannot be explained on the basis of altered classification criteria, as most of the cases had been histologically reviewed (see **Methods**).

The distribution of the 172 cases according to histological type and age is shown in Table II. It is of interest to note that: (i) Most (10/13) of the tumours of epithelial origin were classified as mucinous in type and of these 4 were of borderline malignancy. (ii) Neoplasms of germ cell origin, not surprisingly, represented the great majority (145/172, 84%) of all childhood ovarian cancers. Among them a considerable proportion (14%), was

**Table I** Comparison of original *vs* revised diagnosis—160[a] cases of malignant ovarian tumours in childhood. CCRG, Britain, 1962–78.

| | *Original Diagnosis* | | | | | | | |
|---|---|---|---|---|---|---|---|---|
| | *Dysgerminoma* | *Malignant Teratoma* | *EST/Embryonal Carcinoma* | *Other Germ-Cell* | *Gonado-blastoma* | *Epithelial Carcinomas*[b] | *Sex-Cord Stromal* | *Others* |
| *Revised Diagnosis* | | | | | | | | |
| Dysgerminoma | 43 | — | 1 | — | — | 2 | 3 | — |
| Malignant Teratoma | — | 27 | 1 | 2 | — | 3 | — | — |
| EST* Embryonal Carcinoma | 3 | 2 | 18 | 2 | — | 3 | 1 | — |
| Other Germ-Cell | 1 | 3 | 3 | 11 | 1 | 1 | 1 | — |
| Gonadoblastoma | — | — | — | — | 3 | — | — | — |
| Epithelial Carcinomas | — | — | — | — | — | 12 | — | — |
| Sex-cord Stromal | — | 1 | 1 | — | — | — | 6 | — |
| Others | — | — | 2 | — | — | 1 | 1 | 1 |

[a]Only cases on which some pathological documentation was obtained included (see "**Methods**"). The original diagnoses of the 12 unreviewed cases were as follows:

| | |
|---|---|
| Dysgerminoma | (5) |
| Malignant Teratoma | (3) |
| Embryonal Carcinoma | (1) |
| Choriocarcinoma | (1) |
| Granulosa Cell Tumour | (1) |
| Undifferentiated Carcinoma | (1) |

Of these patients, 7 were diagnosed during 1962–69 and the remaining 5 during 1970–78.

[b]Most of the cases which were re-classified were simply registered as "Carcinoma, not otherwise specified".

composed of two or more cell types: 7 immature teratomas plus EST (in 3 cases with areas of choriocarcinoma), 7 teratomas plus dysgerminomas (and areas of EST, in 3 cases) 2 dysgerminomas plus choriocarcinoma, 1 with EST and 1 with embryonal carcinoma areas. Two cases were unclassifiable mixed germ cell neoplasms. (iii) The cases classified as "others" consisted of 2 patients with primary ovarian Burkitt-like lymphoma (one plus ALL), 2 lipoid cell tumours, and a low-grade sarcoma probably of fibroblast origin.

A total of 11 (6%) neoplasms occurred at $<5$ years of age, 36 (21%) from age 5–9 years and 125 (73%) from 10–14 years (Table II).

While sex-cord stromal tumours appeared more or less uniformly distributed in the various age groups and epithelial tumours were concentrated in the later years, there was a considerable change in the distribution of germ cell tumours over age. More "invasive" neoplasms tended to occur with a higher frequency at a younger age ($<10$ years), while "less malignant" tumours (dysgerminomas) were proportionally more frequent in the later age groups ($\chi_1^2=6.0$; $P=0.01$; Table II).

### *Risk and associated factors*

A total of 24 (14%) patients had one or more congenital defects: these are listed according to cell type, in Table III. Most of them were morphological abnormalities of external or internal genitalia.

As regards family history, a cluster of 2 (and possibly 3) cases of ovarian germ cell neoplasms was found in one family. The mother of a child with a bilateral dysgerminoma had died of an ovarian dysgerminoma plus choriocarcinoma, diagnosed in pregnancy. This patient's grandmother had also had an ovarian tumour, possibly a dysgerminoma. This case had already been reported (Jackson, 1967), but it is now possible to give a rough estimate of the conditional probability of finding two or more members of the same family with an ovarian germ cell tumour. Assuming that the probability of a woman developing a germ cell tumour of the ovary is about 1 in 2000, and that in three generations of the same family there are about 5 women, one should expect to find one further case of germ cell tumour in one out of 500 families of an affected patient, and two further cases in one $6.7\times10^5$ families. It seems therefore extremely unlikely that even this single cluster can have occurred by chance.

As far as risk factors during pregnancy are concerned, among interviewed cases (see **Methods**), none of the considered exposures (X-rays, hormones, sedatives or tranquillizers) or diseases (mainly infections, such as cystitis, pyelitis, chicken

**Table II** Distribution of 172 cases of malignant ovarian tumours in children <15 years registered in Britain, 1962–78 according to histological type and age

| | *Epithelial carcinomas* | | *Sex-cord stromal tumours* | | | *Germ-cell tumours* | | | | | | | *Others* |
|---|---|---|---|---|---|---|---|---|---|---|---|---|---|
| | *Serous/ Undiff.* | *Mucinous* | *Granulosa Cell* | *Sertoli-Leydig Cell* | *Un-Classified* | *Dysgerminomas* | *Malig. Teratomas* | *Endodermal Sinus Tumours* | *Embryonal Carcinomas* | *Chorio Carcinomas* | *Mixed Germ cell* | *Gonado-Blastomas* | |
| Age | | | | | | | | | | | | | |
| <5 | — | — | — | — | 1 | 3 | 4 | 2 | — | — | — | — | 1 |
| 5–9 | — | — | 1 | 2 | 1 | 5 | 11 | 9 | — | — | 5 | 1 | 1 |
| 10–14 | 3 | 10 | 2 | 1 | 1 | 46 | 21 | 15 | 4 | 2 | 15 | 2 | 3 |
| Total | 3 | 10 | 3 | 3 | 3 | 54 | 36 | 26 | 4 | 2 | 20 | 3 | 5 |
| (%) | (1.7) | (5.8) | (1.7) | (1.7) | (1.7) | (31.4) | (20.9) | (15.1) | (2.3) | (1.2) | (11.6) | (1.7) | (2.9) |

**Table III** Distribution of 24 cases of congenital abnormalities according to histological type CCRG, Britain 1962–78

| | *Sex-related abnormalities* | *Mental abnormalities* | *Others* |
|---|---|---|---|
| Gonadoblastomas ($n=3$) | 2 "Streaky ovaries" (1 phenot. intersex) | — | — |
| Dysgerminomas ($n=54$) | 1 "Streaky ovaries"<br>1 Atresia vagina/anus | 3 Epilepsy<br>1 "Mental Abnor." N.O.S. | 2,Minor |
| Malignant teratomas ($n=36$) | 1 "Streaky ovaries"<br>1 Turner syndrome | — | 1 Heart defect (operated)<br>2,Minor |
| Choriocarcinomas ($n=2$) | — | 2 "Mentally Subnormal" | — |
| Endodermal sinus tumours ($n=26$) | 1 Atresia vagina/anus | — | 1,Minor |
| Embryonal carcinomas ($n=4$) | — | — | 1 Horseshoe kidney |
| Mixed germ cell tumours ($n=20$) | 1 Absence of uterine corpus | 1 Epilepsy | 1 Cavernous hemangioma |
| Low grade (fibro)sarcoma ($n=1$) | — | 1 Congenital cataract + Epilepsy | — |

pox, rubella or influenza) was significantly more common among the cases than among their matched controls.

*Stage, size, and site of occurrence*

The distribution according to clinico-pathological stage and histological categories is given in Table IV. A total of 80 cases (47%) were stage I, 5 (3%) stage II, 77 (46%) stage III and 7 (4%) stage IV. Most of the neoplasms were of considerable volume, 91% having a maximum diameter >10 cm.

Eighty-nine tumours (55%) occurred in the right ovary, 62 (38%) in the left ($P=0.03$, exact binomial), 11 (7%) were bilateral. The difference in the site of occurrence was accounted for only by the germ cell tumours (80 right, 49 left, $P=0.008$), other cell types occurring in similar proportions in the two gonads.

*Therapeutic approach*

The outlines of first-line therapeutic approach are summarized in Table V. Surgery was the first obvious treatment step: most of the cases (123, 73%) were treated by unilateral (salpingo) oophorectomy, irrespective of the cell type. Radiotherapy (in most of the cases external-beam radiotherapy to the pelvis or pelvis plus abdomen or plus paraortic nodes) was given to a total of 74 patients, and, not surprisingly, was more commonly used in dysgerminomas. It was also employed as a treatment for recurrence in 27 patients. At least one chemotherapy regimen was given only to a minority of the patients (41/170, 24%), but the proportion of chemotherapy-treated patients increased over more recent years (from 8% in the period 1962–71 to 44% from 1972 onwards).

VAC regimen (vincristine, actinomycin D, cyclophosphamide) was given to 12 patients with germ cell neoplasms, whereas only two patients up to 1978 received any of other newer regimens, such as PVB (cisplatinum, vincristine, bleomycin).

A total of 28 patients received chemotherapy for the treatment of recurrences.

*Survival*

The 10-year survival curves according to individual histological types are presented in Figure 1. Overall, long-term survival was achieved in approximately one in two patients.

Apart from cell type, the only other variable with a clear prognostic significance when the whole case series is considered was clinical stage (Figure 2). No significant diffference was evident according to age at diagnosis in 5-year groups, type of hospital (e.g. teaching *vs* non-teaching), and in all cell types together, size of the neoplasms.

A few specific comments can be made regarding the different tumour types. Among epithelial carcinomas, whose overall 10-year survival was 73%, the three deaths were among mucinous (2 cases) and undifferentiated carcinomas (1 case), all stage III. None of the borderline mucinous adenocarcinomas died of disease, though one of them was stage III at diagnosis.

**Figure 1** Comparative 10-year survival rates according to histological type. 172 cases of malignant ovarian tumours in childhood, CCRG, Britain, 1962–78.

**Table IV** Distribution of 172 cases of malignant ovarian tumours in children <15 years registered in Britain, 1962–78 according to histological type, stage and size

| | *Epithelial Carcinomas* | | *Sex-Cord Stromal Tumours* | | *Germ Cell Tumours* | | | | | | | | | | *Others* | |
|---|---|---|---|---|---|---|---|---|---|---|---|---|---|---|---|---|
| | | | | | *Dysgerminomas* | | *Malignant Teratomas* | | *EST/Embryonal Carcinomas* | | *Other Germ Cell Tumours* | | *Gonadoblastomas* | | | |
| | *No.* | (%) | *No.* | (%) | *No.* | (%) | *No.* | (%) | *No.* | (%) | *No.* | (%) | *No.* | (%) | *No.* | (%) |
| *Clinical Stage* (FIGO)[a] | | | | | | | | | | | | | | | | |
| I | 9 | (75.0) | 4 | (44.4) | 27 | (50.0) | 16 | (45.7) | 7 | (23.3) | 12 | (57.1) | 3 | (100.0) | 2 | (40.0) |
| II | — | — | — | — | 3 | (5.6) | — | — | 1 | (3.3) | 1 | (4.8) | — | — | — | — |
| III | 3 | (25.0) | 5 | (55.6) | 23 | (42.6) | 16 | (45.7) | 21 | (70.0) | 8 | (38.1) | — | — | 1 | (20.0) |
| IV | — | — | — | — | 1 | (1.9) | 3 | (8.6) | 1 | (3.3) | — | — | — | — | 2 | (40.0) |
| Size (cm)[b] | | | | | | | | | | | | | | | | |
| <10 | — | — | 2 | (22.2) | 2 | (4.2) | 1 | (3.6) | 3 | (12.0) | 3 | (15.0) | 1 | (33.3) | 1 | (22.0) |
| 10–14 | 3 | (27.3) | 2 | (22.2) | 25 | (52.1) | 10 | (35.7) | 9 | (36.0) | 5 | (25.0) | — | — | 3 | (60.0) |
| ⩾15 | 8 | (72.7) | 5 | (55.6) | 21 | (43.8) | 17 | (60.7) | 13 | (52.0) | 12 | (60.0) | 2 | (66.7) | 1 | (20.0) |

[a]In 3 cases this information was not available.

[b]In 23 cases this information was not available.

**Table V** Distribution of 172[a] cases of malignant ovarian tumours registered in Britain, 1962–78, according to histological type and first-line therapeutic approach

| | *Epithelial Carcinomas* | | *Sex-cord Stromal tumours* | | *Germ Cell Tumours* | | | | | | | | | | *Others* | |
|---|---|---|---|---|---|---|---|---|---|---|---|---|---|---|---|---|
| | | | | | *Dysgerminomas* | | *Malignant Teratomas* | | *EST/Embryonal Carcinomas* | | *Other Germ Cell Tumours* | | *Gonadoblastomas* | | | |
| | *No.* | (%) | *No.* | (%) | *No.* | (%) | *No.* | (%) | *No.* | (%) | *No.* | (%) | *No.* | (%) | *No.* | (%) |
| Surgery[a] | | | | | | | | | | | | | | | | |
| MSO | 9 | (75.0) | 5 | (55.6) | 39 | (72.2) | 28 | (82.4) | 21 | (70.0) | 17 | (77.3) | 2 | (66.7) | 2 | (40.0) |
| TAHBSO | 2 | (16.7) | — | — | 7 | (13.0) | — | — | 2 | (6.7) | 2 | (9.1) | 1 | (33.3) | 2 | (40.0) |
| Partial removal/ Biopsy only | 1 | (8.3) | 4 | (44.4) | 8 | (14.8) | 6 | (17.6) | 7 | (23.3) | 3 | (13.6) | — | — | 1 | (20.0) |
| Radiotherapy[a] | | | | | | | | | | | | | | | | |
| Yes | 1 | (8.3) | 3 | (33.3) | 35 | (64.8) | 11 | (30.6) | 16 | (55.2) | 7 | (33.3) | 1 | (33.3) | — | — |
| No | 11 | (91.7) | 6 | (66.7) | 19 | (35.2) | 25 | (69.4) | 13 | (44.8) | 14 | (66.7) | 2 | (66.7) | 5 | (100.0) |
| Chemotherapy[b] | | | | | | | | | | | | | | | | |
| Mono-Chem. (Miscellaneous) | 3 | (25.0) | — | — | 4 | (7.5) | 3 | (8.3) | 3 | (10.0) | 5 | (23.8) | — | — | 1 | (20.0) |
| Poly-Chem. (miscellaneous) | — | — | 1 | (11.1) | 2 | (3.4) | — | — | 3 | (10.0) | — | — | — | — | 1 | (20.0) |
| VAC/PVB | — | — | 1 | (11.1) | — | — | 1 | (2.8) | 11 | (36.7) | 2 | (9.5) | — | — | — | — |
| No Chemotherapy | 9 | (75.0) | 7 | (77.8) | 48 | (88.9) | 32 | (88.9) | 13 | (43.3) | 14 | (66.7) | 3 | (100.0) | 3 | (60.0) |

[a]In 3 cases this information was not available.

[b]In two cases this information was not available.

MSO = Monolateral Salpingo-Oophorectomy.

TAHBSO = Total Abdominal Hysterectomy and Bilateral Salpingo-Oophorectomy.

**Figure 2** Comparative 10-year survival rates according to stage (FIGO). 169 cases of malignant ovarian tumours in childhood, CCRG, Britain, 1962–78. $\chi_1^2$ for linear trend = 63.89: $P<0.001$.

In malignant teratomas, prognosis was influenced by clinical stage, degree of histological differentiation and size of the neoplasm (Figure 3). However, these three variables were obviously strongly correlated, and, when they were respectively adjusted with each other only the stage remained statistically significant.

The overall 10-year survival rate among the 20 cases of mixed germ cell tumours was similar to the one for "most malignant" germ cell neoplasms. However, survival was significantly higher in cases containing areas of dysgerminoma (53% achieving long term survival) than in tumours composed only of other cell types (EST, embryonal carcinoma, teratoma, choriocarcinoma. 0/7 patients being alive $\geqslant 3$ years after original diagnosis; $\chi_1^2 = 5.29$, $P = 0.02$).

**Figure 3a** Comparative 10-year survival rates according to stage (FIGO). 35 cases of malignant teratomas in childhood. CCRG, Britain, 1962–78. $\chi_1^2$ for linear trend = 27.08, $P<0.001$; adjusted for grade and size = 11.52, $P<0.001$.

**Figure 3b** Comparative 10-year survival rates according to grade. 30 cases of malignant teratomas in childhood. CCRG, Britain, 1962–78. $\chi_1^2$ for linear trend = 5.38, $P = 0.02$; adjusted for stage and size = 0.28, NS.

**Figure 3c** Comparative 10-year survival rates according to size of the neoplasm. 28 cases of malignant teratomas in childhood. CCRG, Britain, 1962–78. $\chi_1^2$ for linear trend = 3.56 $P = 0.06$; adjusted for stage and grade = 0.08, NS.

The major prognostic indicator among germ cell neoplasms other than dysgerminomas was the use of adequate chemotherapy regimens (VAC/PVB): survival was 86% among 14 treated patients, but only 29% among 77 inadequately treated patients, a statistically significant difference, at the 0.001 level ($\chi_1^2 = 18.90$) when clinical stage is adjusted for (Figure 4).

**Figure 4** Comparative 10-year survival rates according to the use of chemotherapy. 91 cases of non dysgerminomatous germ cell tumours. CCRG, Britain, 1962–78. $\chi_1^2 = 10.70$; $P = 0.001$; adjusted for stage = 18.90, $P < 0.001$.

*Subsequent pregnancy history and long-term follow-up*

Five patients (2 with dysgerminomas, one with mucinous cystadenocarcinoma, one with borderline mucinous cystadenocarcinoma, one with malignant teratoma, all of which were Stage I) had a total of 6 live births. There was no evidence of any abnormality in the offspring.

Only one patient developed a second neoplasm. At age 14 she had received radiotherapy to the pelvis and abdomen for the treatment of a Stage III dysgerminoma, and at age 27 she developed a carcinoma of the pelvic colon (histologically confirmed).

### Discussion

The present study is based on the largest population-based series of malignant ovarian tumours reported to date. Although some information was collected retrospectively, the consistent use of the original hospital records should have prevented the introduction of any important discrepancy in the quality of the data over the 17-year period. Similarly, the revision of 80% of the pathological material (based on sections from each of the several tumour blocks), and of 93% of the pathological reports has resulted in a satisfactory degree of reliability of the classified data. Moreover, the follow-up information can be considered practically complete.

As concerns descriptive epidemiology, this study has not shown any trend in the incidence of (or mortality from) childhood ovarian tumours over the 17-year period considered. This finding is in agreement with the OPCS mortality data (OPCS, 1975) and with similar reports on ovarian tumours in younger children from Scandinavian countries (Lindfors, 1971) and the USA (Li *et al.*, 1973). However, it is at variance with the increase in germ cell tumours (which represent 84% or our series) reported by the Manchester Children's Tumour Registry (Birch *et al.*, 1982), and with the established increase in (germ cell) testicular cancer (Davies, 1981), which however, is mainly evident in older groups.

On the other hand, the present data are in agreement with the Manchester series (Birch *et al.*, 1982) in reporting a noticeable increase in yolk-sac tumours; nonetheless, we do not think it is worth over-stressing the increase, however significant, considering the very low absolute number, and the fact that, when many different sub-groups are considered, it is quite likely that some variations in rates are found simply by chance.

*Histopathological findings*

The histotype distribution if the present series appears in broad agreement with previous work (Groeber, 1963; Lindfors, 1971; Norris & Jensen, 1972; Li *et al.*, 1973; De Palo *et al.*, 1978; Lucraft *et al.*, 1980) germ cell tumours representing by far the majority (>80%) of all childhood ovarian cancers.

The percentage of sex-stromal tumours in our series (5%) is lower than suggested in previous series (Breen & Maxson, 1977). However, their percentage seems likely to be an overestimate, possibly because of misclassification of some germ cell tumours (mainly dysgerminomas) as poorly-differentiated granulosa cell tumours, or to the inclusion of benign tumours of the fibroma-thecoma group.

When germ cell neoplasms are considered, it is worth noting that a careful pathological revision shows that a considerable proportion (14%) of them are composed of two or more cell types (mixed germ cell tumours), a finding which has obvious implications both for treatment and prognosis.

*Risk and associated factors*

The present study has confirmed the association of a high proportion (2 out of 3 cases) of gonado-blastomas with the typical features of gonadal dysgenesis (Scully, 1977). It is also of interest to mention that gross sex-related abnormalities were present in 6/142 (4%) of other germ cell tumours (Table III).

Similarly, the proportion of severe mental or neurological abnormalities (7 affected patients in the whole series) is higher than expected, and no obvious explanation can be given for this association. However, sex-related chromosomal abnormalities are also often related both to abnormal sexual development and to mental handicap.

A high incidence of congenital defects, reflecting a similar pattern, was found in the series described by Fraumeni *et al.* (1973) and Birch *et al.* (1982). The latter postulated a common embryological defect which involves the lower spinal axis and hindgut, resulting in the development of both teratomas and pelvic anomalies.

None of the factors related to mothers' pregnancies we were able to analyse was related to the risk of developing an ovarian tumour in childhood.

Therefore, the only factor which was found to be of importance in some cases is the family history as confirmed by the cluster of two (perhaps three) cases of dysgerminoma in one family (another family cluster of germ cell neoplasms in three sisters and two other related members of the same family was registered in 1979, and is therefore not considered in the present report—see Mann *et al.*, 1982).

That a genetic component represents an important aspect of the risk of developing germ cell neoplasms finds support in three other sets of data: the reported familial occurrence of benign teratomas in women (Hecht *et al.*, 1976); the clustering of germ cell testicular tumours in twins and sibs (Levey & Grabstald, 1975), and their association with HLA DW7 and A10 (De Wolf *et al.*, 1979); and the animal models of germ cell tumours (Graham, 1982).

In addition, it is of interest to comment on the relationship between the age distribution of the various types of germ cell neoplasms and the "invasiveness" of the tumours themselves: "more malignant" tumours occur at significantly lower ages than dysgerminomas; these in turn occur earlier than benign teratomas which are found mainly in adult life. This finding may well provide some support for the view that these tumours have a common origin with, however, different growth patterns apparently proportional to the degree of malignancy of the neoplasm itself.

The occurrence of epithelial cancers in the later age groups (all after menarche) may be taken as a confirmation of their dependence on gynaecological events, and, perhaps, their hormonal correlates (La Vecchia *et al.*, 1983*b*). An individual case worth mentioning in this regard concerns a patient with bilateral serous cystadenocarcinoma associated with long-standing gross obesity. It is now believed (Siiteri, 1978) that adipose tissue is an important site of peripheral aromatization of adrenal androgens to oestrogens.

No case of definite prepuberal epithelial carcinoma has been found in this series. Only two previous reports of such an occurrence are described in the world literature (Hong *et al.*, 1980; Blom & Torkildsen, 1982).

Ten out of 13 epithelial cancers were of mucinous type, which, interestingly enough, has been suggested as deriving from teratomatous origin (Fox *et al.*, 1964; Langley *et al.*, 1972). Among them, 4 were of borderline malignancy, a proportion which, even allowing for the small absolute numbers, seems far higher than in the adult-onset neoplasms. It may therefore be worth looking at these forms as first stages in the same multi-step process of carcinogenesis.

### *Therapeutic approach*

As regards primary treatment, there appears to be little debate on the surgical approach, unilateral (salpingo)-oophorectomy being commonly accepted as the first-line treatment in stage IA disease. A conservative approach in early neoplasms allowed conservation of fertility in a proportion of patients (5 of our cases had a total of 6 normal births).

The introduction of efficacious polychemotherapy regimens certainly represents the most impressive change introduced over the last decade in the management of germ cell neoplasms. Survival in non-dysgerminomatous germ cell neoplasms increased from 29% in untreated patients or patients inadequately treated with chemotherapy to 86% in adequately treated ones (Figure 4). The efficacy of VAC, the first successfully introduced regimen (Smith & Rutledge, 1975) is confirmed in our series, although non-randomized and collected from 10 different hospitals. However, it seems more plausible to assume differences in treatment policies between different hospitals than systematic differences in tumour characteristics (as, for instance, adjustment for stage even increased the value of statistical significance). This may well confirm its efficacy even outside very strict "trial" conditions and, simultaneously, underlines the necessity that every patient with one of these tumour types should be given the opportunity to receive adequate treatment (considering only the period 1972–1978, 31 patients were treated in 29 other hospitals with inadequate, or no medical treatment: among them only 11 are alive).

*Survival*

As far as survival is concerned, apart from the clear relationship with cell type, and the reliability of clinical stage as a prognostic indicator, in the series as a whole no other factor appears relevant. However, some specific observations can be made regarding the different tumour types.

Among the 13 epithelial cancers, the overall 73% survival rate appears higher than the 30–35% commonly reported in adult-onset cancers, and in other small series of epithelial neoplasms in children (Breen & Maxson, 1977). This is probably attributable both to the clinical stage (9/13 patients were Stage I), and to the high proportion of mucinous adenocarcinoma of borderline malignancy in the cases in our series.

On the other hand, the prognosis of our limited series of granulosa cell tumours (all 3 cases died of disease after 2–11 months) appears far inferior than that reported for this cell type (Roth *et al.*, 1979; Young & Scully, 1982). The absolute number of cases reported, however is extremely low, and therefore studies on larger numbers of cases are necessary before knowledge of behaviour of these neoplasms is definitive.

While, not surprisingly, dysgerminomas had a fairly good prognosis (10-year survival, 73%), the broad group of germ cell tumours other than dysgerminoma, however heterogeneous morphologically, seems to exhibit fairly similar overall clinical behaviour. This appears worth stressing in the light of the similarities in general therapeutic approach and chemosensitivity.

It is moreover of interest to make a few distinctions not only between, but also within various cell-types. Among the 36 cases of malignant teratoma, for instance, not only clinical stage, but also tumour size and degree of histological differentiation seemed to have a noteworthy impact on prognosis. This confirms a similar observation originally made by Norris *et al.* (1976). However, when these three variables were adjusted to each other, only the effect of clinical stage on prognosis remained statistically significant.

The prognosis of mixed germ cell tumours (which represent a considerable proportion in the present series) appears extremely poor, particularly when dysgerminomatous areas are not present, and once again underlines the need for extremely careful histopathological analysis and classification as a prerequisite of any rational therapeutic approach.

We are grateful to Dr. L.M. Kinnier Wilson for providing data from the Oxford Survey of Childhood Cancer, to the Marie Curie Memorial Foundation which supports her work, and to all the pathologists who sent the material for review. We thank Mr. C.A. Stiller of the Childhood Cancer Research Group for his help, Mrs. Barbara Crossley and Miss Carol Hermon, of the ICRF Cancer Epidemiology and Clinical Trials Unit for their assistance with data processing and computer programming amd Mrs. Eileen Gunn, Mrs. Jean Pollard and Mrs. Betty Roberts for typing the manuscript.

The work for this paper by Dr. C. La Vecchia was undertaken during the terms of a Research Training Fellowship awarded by the Italian Labour Department and the EEC.

The Childhood Cancer Research Group is supported by the Department of Health and Social Security and the Scottish Home and Health Department.

**References**

BIRCH, J.M., MARSDEN, H.B. & SWINDELL, R. (1982). Pre-natal factors in the origin of germ cell tumours in childhood. *Carcinogenesis*, **3**, 75.

BLOM, G.P. & TORKILDSEN, E.M. (1982). Ovarian cystadenocarcinoma in a 4-year old girl: Report of a case and review of the literature. *Gynecol. Oncol.*, **13**, 242.

BREEN, J.L. & MAXSON, W.S. (1977). Ovarian tumours in children and adolescents. *Clin. Obstet. Gynecol.*, **20**, 607.

DAVIES, J.M. (1981). Testicular cancer in England and Wales: Some epidemiological aspects. *Lancet*, **i**, 928.

DE PALO, G.M., DOCI, R., GASPARINI, M. & FOSSATI-BELLANI, F. (1978). Malignant ovarian neoplasms in childhood. *Tumori*, **64**, 33.

DeWOLF, W.C., LANGE, P.H., EINARSON, M.E. & YUNIS, E.J. (1979). HLA and testicular cancer. *Nature*, **277**, 216.

DRAPER, G.J., BIRCH, J.M., BITHELL, J.F. & 6 others. (1982). Childhood cancer in Britain. Incidence, survival and mortality. *Studies on Medical and Populations Subjects No. 37*. London, HMSO.

FOX, H., KAZZAZ, B. & LANGLEY, F.A. (1964). Argyrophil and argentaffin cells in the female genital tract and in ovarian mucinous cysts. *J. Pathol. Bacteriol.*, **88**, 479.

FRAUMENI, J.F. Jr., LI, F.P. & DALAGER, N. (1973). Teratomas in children: Epidemiologic features. *JNCI*, **51**, 1425.

GRAHAM, C.F. (1982) Initiation of mouse teratomas. In: *Germ Cell Tumours* p. 17 (Eds. Anderson *et al.*) London: Taylor & Francis.

GROEBER, W.R. (1963). Ovarian tumours during infancy and childhood. *Am. J. Obstet. Gynecol.*, **86**, 1021.

HECHT, F., KAISER MC CAW, B. & PATIL, S. (1976). Ovarian teratomas and genetic of germ-cell formation. *Lancet*, **ii**, 1311.

HONG, S.J., LURAIN, J.R., TSUKADA, Y., PIVER, M.S., HUMBERT, J.R. & FREEMAN, A.I. (1980). Cystadenocarcinoma of the ovary in a 4-year-old. Benign transformation during therapy. *Cancer*, **45**, 2227.

JACKSON, S.M. (1967). Ovarian dysgerminoma. *Br. J. Radiol.*, **40**, 459.

LANGLEY, F.A., CUMMINS, P.A. & FOX, H. (1972). An ultrastructural study of mucin secreting epithelia in ovarian neoplasms. *Acta Pathol. Microbiol. Scand.* (*A*), **233**, 76.

LA VECCHIA, C., DRAPER, G.J. & FRANCESCHI, S. (1983*a*). Non-ovarian female genital tract cancers in children in Britain 1962–78: Descriptive epidemiolgy and long term survival. *Cancer*, (in press).

LA VECCHIA, C., FRANCESCHI, S., GALLUS, G., DE CARLI, A., LIBERATI, A. & TOGNONI, G. (1983*b*). "Incessant ovulation" and ovarian cancer: A critical approach. *Int. J. Epidemiol.* (in press).

LEVEY, S. & GRABSTALD, H. (1975). Synchronous testicular tumors in identical twins. *Urology*, **6**, 754.

LI, F.P., FRAUMENI, J.F. Jr. & DALAGER, N. (1973). Ovarian cancers in the young. Epidemiologic observations. *Cancer*, **32**, 969.

LINDFORS, O. (1971). Primary ovarian neoplasms in infants and children. A study of 81 cases diagnosed in Finland and Sweden. *Ann. Chir. Gynaecol. Fenn.*, **177**, 1.

LUCRAFT, H., MANN, J.R. & PEARSON, D. (1980). Malignant ovarian tumours in children. In: *Ovarian Cancer*, (Eds. Newman *et al.*) Oxford. Pergamon.

MANN, J.R., CORKERY, J.J., FISHER, H. & 5 others. (1982). The X-linked form of gonadal dysgenesis. Investigation of a family with a high incidence of gonadal tumours, including cytogenetic, dermatoglyphic and blood group studies and measurement of X–Y antigen. In: *United Kingdom Children's Cancer Study Group*.

NORRIS, H.J. & JENSEN, R.D. (1972). Relative frequency of ovarian neoplasm in children and adolescents. *Cancer*, **30**, 713.

NORRIS, H.J., ZIRKIN, H.J. & BENSON, W.L. (1976). Immature (malignant) teratoma of the ovary. A clinical and pathological study of 58 cases. *Cancer*, **37**, 2359.

OFFICE OF POPULATION CENSUSES AND SURVEYS (OPCS). (1975). Cancer mortality—England and Wales 1911–70. *Studies on Medical and Population Subjects No. 29*. London, HMSO.

OFFICE OF POPULATION CENSUSES AND SURVEYS, (OPCS). (1980, 1981, 1982). Cancer statistics registrations—Cases of diagnosed cancer registered in England and Wales, 1975, 1976, 1977. London, HMSO.

PETO, R., PIKE, M.C., ARMITAGE, P. & 7 others. (1977). Design and analysis of randomized clinical trials requiring prolonged observation of each patient. II. Analysis and examples. *Br. J. Cancer*, **35**, 1.

ROBBOY, S.J. & SCULLY, R.E. (1970). Ovarian teratoma with glial implants on the peritoneum. An analysis of 12 cases. *Human Pathol.*, **1**, 643.

ROTH, L.M., NICHOLAS, T.R. & EHRLICH, C.E. (1979). Juvenile granulosa cell tumor. A clinicopathologic study of three cases with ultrastructural observations. *Cancer*, **44**, 2194.

SCULLY, R.E. (1977). Ovarian tumours. A review. *Am. J. Pathol.*, **87**, 686.

SIITERI, P.K. (1978). Steroid hormones and endometrial cancer. *Cancer Res.*, **38**, 4360.

SMITH, J.P. & RUTLEDGE, F. (1975). Advances in chemotherapy for gynecologic cancer. *Cancer*, **36**, 669.

YOUNG, R.H. & SCULLY, R.E. (1982). Ovarian sex cord-stromal tumors: Recent progress. *Int. J. Gynecol. Pathol.*, **1**, 101.